# High‐yield, plant‐based production of an antimicrobial peptide with potent activity in a mouse model

**DOI:** 10.1111/pbi.14460

**Published:** 2024-09-12

**Authors:** Shahid Chaudhary, Zahir Ali, Aarón Pantoja‐Angles, Sherin Abdelrahman, Cynthia Olivia Baldelamar Juárez, Gundra Sivakrishna Rao, Pei‐Ying Hong, Charlotte Hauser, Magdy Mahfouz

**Affiliations:** ^1^ Laboratory for Genome Engineering and Synthetic Biology, Division of Biological Sciences King Abdullah University of Science and Technology (KAUST) Thuwal Jeddah Saudi Arabia; ^2^ Laboratory for Nanomedicine, Division of Biological and Environmental Science and Engineering King Abdullah University of Science and Technology (KAUST) Thuwal Jeddah Saudi Arabia; ^3^ Computational Bioscience Research Center King Abdullah University of Science and Technology (KAUST) Thuwal Jeddah Saudi Arabia; ^4^ Red Sea Research Center King Abdullah University of Science and Technology (KAUST) Thuwal Jeddah Saudi Arabia; ^5^ Water Desalination and Reuse Center, Division of Biological Sciences and Engineering King Abdullah University of Science and Technology (KAUST) Thuwal Jeddah Saudi Arabia

**Keywords:** antimicrobial peptides, biomanufacturing, pre‐clinical model, plant biosynthetic chassis

## Abstract

Plants offer a promising chassis for the large‐scale, cost‐effective production of diverse therapeutics, including antimicrobial peptides (AMPs). However, key advances will reduce production costs, including simplifying the downstream processing and purification steps. Here, using *Nicotiana benthamiana* plants, we present an improved modular design that enables AMPs to be secreted via the endomembrane system and sequestered in an extracellular compartment, the apoplast. Additionally, we translationally fused an AMP to a mutated small ubiquitin‐like modifier sequence, thereby enhancing peptide yield and solubilizing the peptide with minimal aggregation and reduced occurrence of necrotic lesions in the plant. This strategy resulted in substantial peptide accumulation, reaching around 2.9 mg AMP per 20 g fresh weight of leaf tissue. Furthermore, the purified AMP demonstrated low collateral toxicity in primary human skin cells, killed pathogenic bacteria by permeabilizing the membrane and exhibited anti‐infective efficacy in a preclinical mouse (*Mus musculus*) model system, reducing bacterial loads by up to three orders of magnitude. A base‐case techno‐economic analysis demonstrated the economic advantages and scalability of our plant‐based platform. We envision that our work can establish plants as efficient bioreactors for producing preclinical‐grade AMPs at a commercial scale, with the potential for clinical applications.

## Introduction

Clinically severe or life‐threatening drug‐resistant bacterial infections present a significant global health challenge (Collaborators, 2022). Currently, the main approach to combat such infections involves antibiotics (Czaplewski *et al*., 2016); however, the increasing prevalence of drug‐resistant isolates, observed in both hospital settings and the community (Mulvey and Simor, 2009), underscores the necessity for innovative antimicrobial agents utilizing modes of action distinct from those of current antibiotics (Miethke *et al*., 2021).

As an alternative to conventional antibiotics, host defence peptides, also known as antimicrobial peptides (AMPs), are small, amphipathic peptides of 10–50 amino acids (aa), typically characterized by a positive charge ranging from +2 to +9 (Haney and Hancock, 2013). AMPs have anti‐inflammatory and anti‐infective activities (Mookherjee *et al*., 2020), as demonstrated in preclinical studies using animal models of mouse (*Mus musculus*) (Achtman *et al*., 2012; Nijnik *et al*., 2010; Piyadasa *et al*., 2018; Scott *et al*., 2007), rat (*Rattus norvegicus*) (Brakel *et al*., 2019; Park *et al*., 2022), dog (*Canis lupus familiaris*) (Brakel *et al*., 2019) and macaque (*Macaca fascicularis*) (Park *et al*., 2022). However, AMPs are currently expensive to manufacture and therefore not commonly used in resource‐poor areas of the world. Strategies for simplifying the downstream processing and purification steps will decrease production costs and thus increase the potential for AMP therapy to combat multi‐drug‐resistant infections in developed and developing countries.

Many of the AMPs that have been tested in preclinical trials are peptides produced using solid‐phase peptide synthesis (SPPS). However, the production of 1 kg of peptide yields between 3000 and 15 000 kg of hazardous waste (Kopach, 2018) containing solvents that are currently under consideration for restriction (Martin *et al*., 2021). Alternatively, genetically engineered *Escherichia coli* cells have been used for high‐titre AMP production (Gaglione *et al*., 2019), but challenges persist, including endotoxin generation (Merlin *et al*., 2014) and the high operational costs of bioreactors (Buyel, 2018). Consequently, bacterially produced AMPs have primarily been confined to animal use. In another instance, cells of the yeast *Pichia pastoris* were engineered to produce grams of AMPs per litre of ferment supernatant (Cao *et al*., 2018; Liu *et al*., 2020; Shen *et al*., 2021), leading to Phase II clinical trials conducted by Hexima Limited for topical treatment of onychomycosis (van der Weerden *et al*., 2023). However, the high associated costs ultimately resulted in the discontinuation of the trials (https://hexima.com.au; Australia).

As an alternative to SPPS and microbial bioreactors, plants are gaining momentum as favourable platforms for producing recombinant antimicrobials for topical microbicides (O'Keefe *et al*., 2009; Ramessar *et al*., 2008). For example, plants are amenable to large‐scale production (Buyel *et al*., 2017), have low production costs (McNulty *et al*., 2020), and are generally recognized as safe by regulators (Nomad Biosciences, Halle, Germany; GRN 593 and GRN 676). Plant platforms also produce less endotoxins compared to bacterial chassis (Merlin *et al*., 2014) and generate minimal liquid waste (Buyel, 2018). Additionally, plant systems can readily comply with good manufacturing practices‐based production (Ma *et al*., 2015). In plants, peptides can be expressed from nuclear genes or from the plastid genome; plastid engineering can increase the production of peptides expressed from the many copies of the plastid genome present in each plastid (Holaskova *et al*., 2015). Besides, plant platform also facilitates secretion of therapeutics into a clean apoplastic space, similar to *Saccharomyces cerevisiae* and *Cricetulus griseus* cells (Novo Nordisk, Denmark), as exemplified by the establishment of plant‐based spinoff companies focused on apoplast purification, such as Fraunhofer (Fischer *et al*., 1999; Schillberg *et al*., 1999), Icon Genetics (Gils *et al*., 2005), Nomad Biosciences (Hahn *et al*., 2015) and Large Scale Biology Corporation (McCormick *et al*., 1999, 2003, 2008). Therefore, further developing a scalable, streamlined workflow for apoplast‐based production of challenging‐to‐produce and therapeutically important peptides, such as AMPs, can effectively address concerns with sustainability and cost in the peptide industry (Chaudhary *et al*., 2024).

There are numerous aspects of plant‐based AMP production that still need optimization. For example, AMPs can damage plant cell membranes (Hoelscher *et al*., 2022; Scotti *et al*., 2015), as they do in bacteria (Lazzaro *et al*., 2020; Nguyen *et al*., 2011). Moreover, plant systems work better for expressing anionic peptides compared with cationic ones (Ghidey *et al*., 2020). The small size of AMPs can lead to their degradation by proteases (Florack *et al*., 1995; Moberg *et al*., 2003), and the presence of hydrophobic domains in AMPs can pose challenges for organelle‐based expression (Scotti *et al*., 2015). Most AMPs undergoing clinical trials are highly cationic (Hancock and Sahl, 2006), hydrophobic (Wang *et al*., 2021) and short in length (Deslouches *et al*., 2013; Ong *et al*., 2014). AMPs displaying strong antimicrobial activity have also shown high toxicity towards plant cells; consequently, some studies have expressed shorter AMPs with moderate activity, including those that have been modified to decrease antimicrobial activity (Osusky *et al*., 2000). The 13‐aa‐long indolicidin (Bhargava *et al*., 2007), 17 aa D4E1 (Rajasekaran *et al*., 2005) and 19 aa MasrA3 (Osusky *et al*., 2004) are among the shortest AMPs expressed in plants; however, these are used primarily for enhancing plant disease resistance rather than for clinical applications. Additionally, downstream processing accounts for 80% of the total production costs in plant‐based protein production (Buyel, 2015).

Previous studies on plant‐based peptide production have shown some successes and defined some approaches to address these challenges. One study used plastid engineering in *Nicotiana tabacum* to express the AMPs retrocyclin‐101 (18 aa) and protegrin‐1 (18 aa), resulting in high accumulation, with AMP yields of 38% and 26% of total soluble protein, respectively (Lee *et al*., 2011). Additionally, these AMPs killed *Streptococcus mutans*, a Gram‐positive coccus found in the oral cavity and prevented biofilm formation after a single topical application to a tooth‐mimetic surface (Liu *et al*., 2016). Recently, a similar plastid platform in *N. tabacum* was used to express a plethora of AMPs (18–46 aa) with a maximum yield of 56 μg per gram fresh weight of leaf material (Hoelscher *et al*., 2022). However, chloroplast transformation is challenging and takes several months due to the number of rounds of selection needed (Verma and Daniell, 2007). Alternatively, a transient expression platform, which takes only a few weeks, has also been used to express protegrin‐1 in *N. tabacum*, albeit with a very low yield (Patiño‐Rodríguez *et al*., 2013). Another study reported unprecedented enhancement in the yield of anionic AMPs (41–49 aa) to 373–563 μg of plant tissue, equaling yield to that of an *E. coli* culture, using the simple and inexpensive elastin‐like polypeptides (ELP) tag, averting the necessity to release the peptide, and displaying high potency against bacteria (Ghidey *et al*., 2020). Although the retained tag did not hinder the peptide's function in the plant, introducing additional amino acids could potentially impede the AMP's function and might face challenges during FDA clinical translation approval.

**Figure 1:**
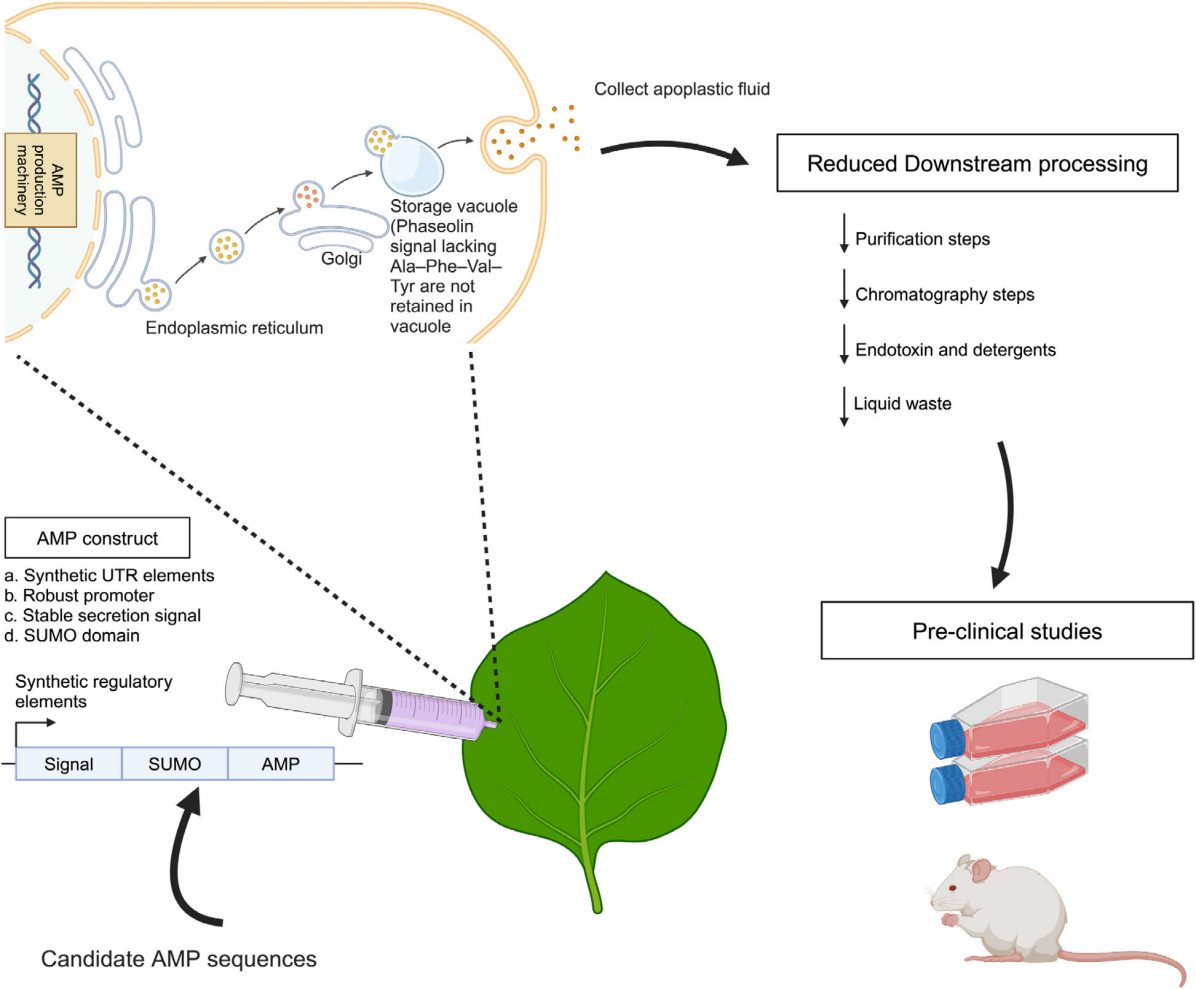
Plant‐based platform that enables large‐scale production of AMPs with potential for clinical applications. Our developed platform provides a rapid means for efficient, large‐scale production of AMPs with reduced downstream processing. Any AMP candidate, regardless of its hydrophobicity, charge and size, can be expressed and produced in less than 2 weeks. The purified AMPs are of pre‐clinical‐testing grade. Additional information about the gene expression constructs and methods used for transient expression can be found in the Methods section.

Previously, we reported that recombinant, post‐translationally amidated AMPs could be expressed and purified with high efficiency in *Nicotiana benthamiana* plants (Chaudhary *et al*., 2023; Chaudhary and Mahfouz, 2024). However, there remains a need for a platform that enables large‐scale production of AMPs with reduced downstream processing. Here, we describe a platform for the production of the clinical immune‐defence regulator 1002 peptide (AMP1, VQRWLIVWRIRKG) via transient expression of an apoplast‐localized AMP in *N. benthamiana* plants. This approach reduced the plant toxicity associated with high levels of AMP and decreased downstream processing costs (Figure 1). Techno‐economic modelling demonstrated the economic feasibility of the platform. Overall, our results highlight the exceptional flexibility of plant‐based production platforms for the large‐scale production of short, pre‐clinical‐grade peptides, with further potential for application in clinical studies.

## Materials and methods

### Cloning of phaseolin‐fused AMP for expression

The high recombinant expression associated with CPMV (pHREAC) expression vector, which contains plant‐specific enhancer elements, a 35S promoter, a synthetic 5′ untranslated region (UTR)‐appended at the 5′ designated with number ‘0’ (5S0), and 3′UTR of *Cowpea mosaic* virus (CPMV) along with nopaline synthase (nos) terminator sequences, was obtained from Addgene (plasmid number 134908). The AMP1 coding sequence was fused with the apoplast secretory signal peptide from phaseolin (Δ418) from a common bean (*Phaseolus vulgaris*). This signal lacks the four‐amino acid hydrophobic pro‐peptide at the C terminus. This fusion construct was directly ordered as a dsDNA gene fragment consisting of phaseolin (Δ418; NCBI protein ID GenBank: AAA99534.1), a twin Strep‐tag sequence, an HA epitope, a GGGGSGGGGS linker, a mutated Small Ubiquitin‐like Modifier domain from *Brachypodium distachyon* (bdSUMO^Eu1^) module fused to the N terminus of the AMP. The entire dsDNA gene fragment was put under the control of the intermediate pHREAC expression vector promoter using Golden Gate assembly. This involved initially digesting the pHREAC vector with *Bsm*BI and *Bsa*I enzymes to remove the intervening 5S0 sequences. Overlapping oligos containing 5′ *Bsm*BI and 3′ *Bsa*I overhangs were then used to reinsert the 5S0 sequences [a synthetic UTR‐appended at the 5′ designated with the number ‘0’], and the gBlock fragment was digested with *Bsa*I to generate compatible overhangs with the 3′ 5S0 sequence and the 3′UTR (CPMV) followed by nos terminator sequences in the pHREAC vector. The full‐length phaseolin‐fused AMPs, including enhancer, 5′ and 3′ elements and nos terminator were PCR amplified using primers containing an *Asc*I restriction site on both (5′ and 3′) ends and subsequently inserted into an *Asc*I‐digested final‐destination vector, the modified Δ35pMDC43 vector, which lacks all regulatory sequences and only contains the aminoglycoside phosphotransferase gene, providing resistance to kanamycin within the T‐DNA. All plasmids were verified by sequencing (KAUST Bioscience Core Lab). All oligonucleotides were purchased from Integrated DNA Technologies (IDT, Leuven, Belgium) and were HPLC‐purified by the manufacturer. Sequences of the oligonucleotides are listed in Data S1.

### Agroinfiltration of *N. benthamiana* plants

*Nicotiana benthamiana* plants were cultivated in greenhouses and were kept at 23 to 25 °C. The binary plasmids Δ35pMDC‐AMP1 and p35S‐p19 were transformed individually into *Agrobacterium tumefaciens* (GV1301) cells using a Bio‐Rad Gene Pulser XCell Electroporation System (Bio‐Rad Laboratories, Hercules, CA). Glycerol stocks of strains were used to inoculate litre of Luria‐Bertani media containing kanamycin (50 mg/L), gentamicin (30 mg/L) and rifampicin (25 mg/L) and pelleted by centrifugation at 2000 ***g***. Following resuspension in MMA (10 mM MES (2‐[N‐morpholino] ethanesulfonic acid) pH 5.7, 10 mM MgCl_2_, 100 μM acetosyringone) to an OD_600_ of 0.2 and a 2–4 h incubation at ambient temperature, suspensions were infiltrated into the leaves of 5 weeks post‐germination soil‐grown *N. benthamiana* using a needleless syringe, and infected plants were left for 5 days. In co‐infiltration experiments, vectors were always infiltrated into *N. benthamina* leaves at equal densities.

### Apoplast wash, recovery and peptide purification

Five days post‐infiltrated leaves were collected, rinsed with cold Milli‐Q water to eliminate any settled dust on their surfaces, and subsequently submerged in a chilled extraction (100 mM Tris HCl pH 8.0, 150 mM NaCl) containing 0.02% [v/v] Silwet L‐77 and complete EDTA‐free protease inhibitor cocktail tablet (Roche, UK), and vacuum‐infiltrated for at least 5 min. The residual buffer was wiped out, multiple infiltrated leaves were wrapped around sterile 10‐mL pipette tips separated by a thin layer of parafilm and multiple sets of arranged leaves were placed in a 1‐L polypropylene bottle (Beckman Coulter) and centrifuged at 250 ***g*** for 20 min at 4 °C. The apoplast‐washed fluid was recovered, further centrifuged at 12 000 ***g***, and filtered through a Nalgene disposable bottle‐top filter with a 0.45‐μm membrane (Thermo Fisher Scientific). The clarified supernatant was applied to 1 mL of *Strep*‐Tactin Superflow resin (Qiagen, Hilden, Germany) and incubated at 4 °C with gentle rotation for 2 h, followed by resin washes with buffer (100 mM Tris–HCl pH 8.0, 150 mM NaCl, 3 mM DTT) to remove loosely bound proteins. After washing the resin, it was immediately resuspended in a small ubiquitin‐like modifier (SUMO) digestion buffer (45 mM Tris–HCl pH 8, 150 mM NaCl, 10 mM DTT). Recombinant AMPs were released under native form by overnight cleavage with purified sentrin‐specific protease (SENP^EuH^) as described previously (Chaudhary *et al*., 2023) at 4 °C under gentle rotation. The released peptide was immediately lyophilized, resuspended in reverse‐phase buffer (5% [v/v] HPLC‐grade acetonitrile and 0.01 M hydrochloric acid) and then injected into the column via the automated injector present in the 1260 Infinity HPLC system. The semi‐preparative C8 column was 9.4 × 250 mm with a 5 μm particle size (Agilent Technologies). Mobile phase A (5% [v/v] HPLC‐grade acetonitrile and 0.01 M hydrochloric acid) and mobile phase B (80% [v/v] HPLC‐grade acetonitrile and 0.01 M hydrochloric acid) were used; fractions were collected after every 2 min at a flow rate of 2 mL min^−1^. The fractions absorbing at dual wavelengths (215/280 nm) were analysed on 12% Tricine–SDS–PAGE, pooled, lyophilized (Labconco CentriVap −105 °C Trap Temperature), and kept at −20 °C until further use.

### Mass spectrometry sequencing using Orbitrap fusion Lumos

The lyophilized peptide was desalted using Sep‐Pak C18 cartridges (Waters), eluted using buffer (0.1% [v/v] trifluoroacetic acid and 75% [v/v] HPLC‐grade acetonitrile), dried and resuspended in 0.1% [v/v] formic acid. The peptide was injected in an Orbitrap Fusion Lumos mass spectrometer (Thermo Scientific) coupled with an UltiMate 3000 UHPLC system (Thermo Scientific) for data‐dependent acquisition (DDA) analysis. In DDA mode, a comprehensive MS scan covering the 350–1400 m/z range was conducted in the Orbitrap at a resolution of 120 000 (at 200 m/z) in profile mode. The interval between master scans was set to 3 s, with the radio frequency lens at 30%. Ion accumulation time was capped at 100 ms with a target value of 4e5. Monoisotopic peak determination of peptide was enabled, and ions within a 1.6 m/z isolation window, surpassing an intensity threshold of 5 e4 and carrying charges from 2+ to 5+, were chosen for fragmentation via higher energy collision dissociation at 30% energy. These peptides were subsequently excluded dynamically after 1 event for 10 s with a mass tolerance of 10 ppm. The option to inject ions for all available parallelizable times was chosen to enhance the quality of MS/MS spectral data. The MS/MS spectra were acquired in Orbitrap in centroid mode, with the first fixed mass set at 100 (m/z) and a resolution of 30 000 (at 200 m/z). The mass spectrometry data files from the Orbitrap Fusion (.raw file) were converted into mgf files using msConvert before performing a database search with the MASCOT software (v2.8, Matrix Sciences Ltd., London, UK) to identify peptides. The search settings included a fragment tolerance of 0.6 Da, a precursor tolerance of 5 ppm, fixed carbamidomethyl modification on cysteine, variable oxidation modification on methionine and allowance for up to two missed trypsin cleavages. Peptides with an expectation level below 0.05 were selected, ensuring a false discovery rate of 2.5%.

### Minimal inhibitory concentration and 1‐*N*‐phenylnaphthylamine uptake assays

The MIC values of plant‐purified AMP1 were determined using the broth microdilution method in cation‐adjusted Mueller–Hinton broth (Ca‐MHB) as described previously (Chaudhary *et al*., 2023). The synthetic AMP1 (purchased from a commercial source, GenScript) was used as a control. To explore whether the purified peptide retains permeability of the outer membrane of bacterial cells, 1‐*N*‐phenylnaphthylamine (NPN) uptake assay was performed based on the Hancock and Wong method (Hancock and Wong, 1984). NPN is an uncharged, hydrophobic fluorescent probe that exhibits weaker fluorescence in an aqueous environment and stronger fluorescence within the hydrophobic interior of membranes. Upon membrane perturbation, NPN can access the hydrophobic environment within the membrane, causing it to emit intense fluorescence. Bacterial cells were grown to an OD at 600 nm of 0.5, centrifuged and diluted in a 5 mM sterile buffer solution of *N*‐2‐hydroxyethylpiperazine‐*N′*‐2‐ethanesulfonic acid (HEPES, pH 7.2) containing 5 mM glucose for 0.4 OD mL^−1^. The peptide solution, prepared in endotoxin‐free sterile water, was added to a 96‐well plate at its respective minimum inhibitory concentration. Ten microlitres of 0.5 mM solution of NPN (Sigma Aldrich, Cat. no. 104043) was added to the plate containing peptide solution in the dark to prevent photophysical decomposition. Ninety microlitres of the bacterial solution, previously prepared in HEPES, was added to the plate in the dark and immediate readings were taken, followed by measurements every minute for 30 min with the fluorescence emission intensity recorded (λexc = 340 nm, λem = 405 nm) using a TECAN Infinite 200 PRO series (Tecan i‐control 2; 2.0.10.0, Austria, GmbH). All experiments, including the controls, were performed in triplicates. The controls included HEPES solution alone, HEPES solution with NPN, HEPES solution with respective bacteria, and HEPES solution containing both bacteria and NPN.

### Limulus amebocyte lysate endotoxin measurement

Endotoxin levels in the purified peptide were determined using the Pierce Chromogenic Endotoxin Quantitation Kit (Thermo Fisher Scientific, Rockford, Cat. no. A39552). The chromogenic Limulus amebocyte lysate assay replaces the coagulogen protein with a chromogenic substrate, consisting of a small peptide linked to p‐nitroaniline (a chromophore), which can be cleaved by the clotting enzyme. The yellow colour produced by substrate cleavage, measured spectrophotometrically at 405 nm, directly correlates with the quantity of endotoxin present in the sample. Pyrogen‐free consumables, such as pipette‐tips, reservoirs and microplates, were used to avoid false positives. Endotoxin standards were prepared as per the recommendations of the manufacturer in the range of 0–1 EU mL^−1^. Samples were assayed in duplicate at 1:1 and 1:5 dilutions and measurements were performed according to the manufacturer's instructions.

### Proteins and peptide analysis

Protein preparations were separated on NuPAGE gels (Thermo Fisher Scientific) and stained with Coomassie blue to detect proteins or alternatively, transferred to a polyvinylidene difluoride membrane with a pore size of 0.45 μm (Amersham Hybond‐P; GE Healthcare Life Sciences) for immunodetection of proteins, as described previously (Chaudhary *et al*., 2023). Peptides were separated on 12% Tricine–SDS–PAGE gels in parallel with the Spectra Multicolor Low Range Protein Ladder (Thermo Fisher Scientific) and stained with Coomassie brilliant blue G‐250 solution, containing 40% methanol and 4% formaldehyde. Peptide concentrations were quantified with the Pierce BCA Protein Assay Kit (Thermo Fisher Scientific, Cat no. 23227) and measured with a microplate reader Infinite 200 PRO series (Tecan i‐control 2; Austria, GmbH) using a bovine serum albumin dilution series as standard.

### Cytotoxicity assays on primary human dermal fibroblasts

Primary human dermal fibroblasts (HDFs) isolated from neonatal foreskin were purchased from Gibco, under the catalogue number C0045C. HDFs were cultured using DMEM cell culture media (Gibco) containing heat‐inactivated 10% fetal bovine serum and 1% penicillin/streptomycin within T75 flasks until they reached 90% confluency. Cells were seeded in 96‐well plates at densities of 4000 cells/well for ATP release assays and 20 000 cells/well for the LIVE/DEAD viability assays, as well as immunostaining experiments.

The biocompatibility of the plant‐purified peptide AMP1 was tested on HDFs at 30 and 50 μg/mL after 12‐ and 24‐h treatments, using the LIVE/DEAD Viability/Cytotoxicity Kit for mammalian cells (Thermo Fisher Scientific, Cat no. L3224). In this assay, viable cells emit green fluorescence through their interaction with Calcein (494/517 nm), while non‐viable cells exhibit red fluorescence caused by the binding of ethidium homodimer‐1 (EthD‐1) to the DNA of cells with compromised cell membranes. Briefly, the culture media was substituted with a solution containing Calcein for detecting viable cells and EthD‐1 for detecting dead cells, diluted in 1× DPBS following the instructions provided by the manufacturer. The treated cell culture plates were kept at room temperature for 30 min. The identification and visualization of the cells were done using a fluorescence microscope (Evos M7000, Thermo Fisher Scientific).

Additionally, the CellTiter‐Glo 3D Cell Viability Assay (Promega, Cat no. G968A) was used to assess cellular metabolic activity based on ATP release. Briefly, an equivalent amount of CellTiter‐Glo reagent, relative to the volume of the media, was added to each well. The contents were mixed by pipetting up and down 8–10 times and then incubated for 30 min. After incubation, a BMG Labtech plate reader was used to read the luminescence signal.

To further understand the peptide's influence on cell morphology, the organization and structure of actin fibres were examined after staining with rhodamine‐phalloidin (Invitrogen, Thermo Fisher Scientific, Cat no. R415). Initially, cells were fixed in 4% paraformaldehyde (Santa Cruz Biotechnology, Cat no. sc‐281692) for 30 min at room temperature, followed by washing with 1× DPBS. Subsequently, the cells were permeabilized with a solution containing 0.5% Triton X‐100, 3 mM of MgCl_2_ and 300 mM sucrose, mixed in 1× DPBS. This buffer was added to all the cell culture plate wells, and the cells were kept at ambient temperature for 5 min. To prevent non‐specific interactions, a blocking buffer composed of 0.1% Tween‐20, 5% Fetal Bovine Serum and 0.02% sodium azide in 1× DPBS was added, and the cells were left to incubate for another 30 min at room temperature. For staining the actin filaments, rhodamine‐phalloidin was diluted to 5 μL per 200 μL of 1× DPBS (1:40), then added to the HDFs and incubated for an hour at room temperature. Following this, the cells were rinsed with 1× DPBS and then exposed to 4′,6‐diamidino‐2‐phenylindole (DAPI), which was diluted to 1:2000 in 1x DPBS for nucleus counterstaining. Cells were imaged using a ZEISS confocal microscope (ZEISS LSM 710 Inverted Confocal Microscope, Germany).

### Bioactivity against the murine cutaneous abscess model

All procedures involving animal experiments were carried out in accordance with the approved protocol by the Institutional Animal Care and Use Committee (IACUC; certificate no: 22IACUC08) at King Abdullah University of Science and Technology (KAUST) and regulated by the Saudi National Committee of Bio‐Ethics (NCBE, registration no: HAP‐02‐J‐042). The use of clinical bacterial pathogens in this study was approved by the KAUST Institutional Biosafety and Bioethics Committee (IBEC no: 22IBEC006). All experiments involving mice (*M. musculus*), including the cultivation of pathogens, were carried out in a highly controlled BSL2 condition. To maintain the BSL2 housing condition, mice were kept in individually ventilated cage systems, which were maintained at negative pressure. All mice used in this study were female outbred CD‐1 mice (Charles River, UK) and 7 weeks old. Mice were kept on an alfalfa‐free diet a week prior to the experiment to reduce the levels of background produced by chlorophyll. Mice were given two doses of the immunosuppressant drug cyclophosphamide (Sigma Aldrich, Cat no. C0768) delivered subcutaneously in 0.9% sterile saline (200 mg Kg^−1^ on day 1 and 150 mg Kg^−1^ on day 4). On day 5, mice were initially anaesthetised with 5% isoflurane (Baxter) in oxygen as carrier gas using the VetFlo single‐channel anaesthesia system, and subsequently the level of isoflurane was reduced to 2% and maintained throughout the entire experiment. The anaesthetic was delivered using a calibrated vaporizer (Model VIP 3000, Midmark Corp, OH) with a carrier gas composed of 20% O_2_ and 80% N_2_O. The abdominal flank of each mouse was shaved using an electric razor and 50 μL of a mid‐logarithmic growth‐phase culture of bioluminescent methicillin‐resistant *Staphylococcus aureus* (MRSA) USA300, containing 1 × 10^7^ CFU, was injected subcutaneously in the abdominal flank to create an abscess. The MRSA USA300 strain used was kindly provided by Dr. Roger Plaut (Centre of Biologics and Research, US Food and Drug administration) and harbours plasmid pRP1195 stably integrated into the chromosome, containing genes for the lux operon (luxC, luxD, luxA, luxB, luxE from *Photorhabdus luminescens*), which confers luminescence. One hour after inoculation, about 7 mg/kg of the peptide in 5% dextrose was injected subcutaneously into the infected area (intra‐abscess injection). As controls, the vehicle 5% dextrose (Sigma Aldrich, Cat no. DX0145), synthetic AMP1 (purchased from a commercial source GenScript) and Vancomycin hydrochloride (Sigma Aldrich, Cat no. PHR1732) were used.

Seventy‐two hours after infection, mice were imaged using the IVIS Spectrum *in vivo* imaging system (PerkinElmer Inc., MA) and images were acquired using a CCD camera with the following parameters: binning = medium; f/stop 4. Bioluminescence intensity was measured and analysed by the Living Image software 3.2 (Caliper Life Sciences, MA, US). The bioluminescence generated from the bacteria was quantified in values of total flux (photons/s). Once the images were acquired, the mice were euthanized, the area of infected skin was excised, homogenized in sterile PBS, and serially diluted for CFU quantification.

### Techno‐economic analysis

We conducted a techno‐economic analysis, as described in our previous study, to determine whether apoplast secretion of AMPs reduces the burden on downstream processing. The base case scenario considers 10 000 kg *N. benthamiana* plant fresh weight (FW) containing 2 kg AMP with an expression level of 200 mg per kg plant FW. Electricity, labor and charges for hot and cold water were calculated according to the local standards in Saudi Arabia as described previously.

## Results and discussion

### Δ418 tagging leads to active apoplast secretion of SUMO‐AMP1

To optimize plant‐based AMP production, we tested the production and apoplast secretion of AMPs in *N. benthamiana*. Apoplast secretion allows for the purification of proteins free from cellular impurities, reduces the necessity for extensive downstream processing and is typically favoured in industrial settings. However, directing proteins, mainly antimicrobials, to the apoplast often has detrimental effects on plant cells since the AMPs can interact with cellular membranes, resulting in necrosis associated with hypersensitive response‐like cell death (Kim *et al*., 2018). Therefore, we aimed to develop an expression system that avoids cell death in the plant and achieves high levels of secreted target protein.

To escort AMP1 into the apoplast, we used a plant‐based apoplast secretory signal peptide from phaseolin, a storage protein from the common bean (*Phaseolus vulgaris*). In plants, phaseolin (NCBI protein ID GenBank: AAA99534.1) is targeted to storage vacuoles, but introducing a stop codon after residue 417 (which results in a protein lacking the last four amino acid residues, Ala‐Phe‐Val‐Tyr) to generate a variant (hereafter referred to as Δ418) abolishes vacuolar targeting, causing the protein to be secreted into the apoplast (Frigerio *et al*., 2001). The stable secretion signal in phaseolin also leads to high‐level storage in membrane‐bound organelles or secretion into the media when introduced into oocyte cells from *Xenopus laevis* (Bassuner *et al*., 1983), insect cells (Sf9) from the moth *Spodoptera frugiperda* (Bustos *et al*., 1988) or in *S. cerevisiae* (Kaufmann *et al*., 2021).

**Figure 2:**
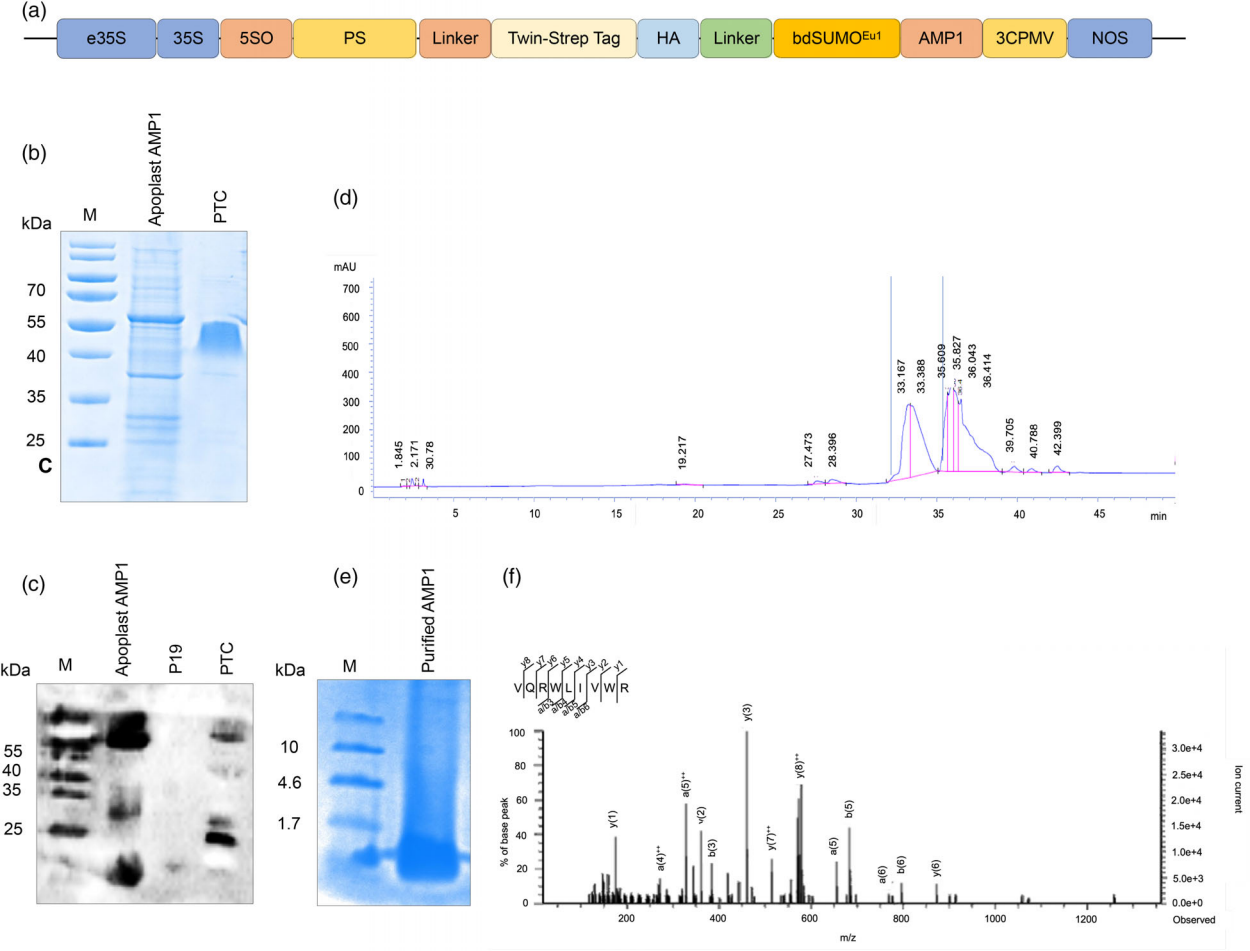
Expression of AMP1 for apoplast targeting in *N. benthamiana*. (a) Schematic representation of the AMP1 expression cassette designed for apoplast sequestration using the backbone of the pMDC43 vector. e35S, enhancer region of 35S promoter; 35S, cauliflower mosaic virus 35S promoter; a synthetic 5′UTR namely the 5S0 [a synthetic untranslated region (UTR)‐appended at the 5′ designated with the number ‘0’] and 3′CPMV (*Cowpea mosaic* virus UTR); PS, signal sequence sourced from phaseolin from *Phaseolus vulgaris* for apoplast secretion; bdSUMO^Eu1^, mutated SUMO domain; HA, hemagglutinin epitope; *nos*, nopaline synthase terminator. (b) Analysis of apoplast wash fluid with SDS–PAGE. The apoplast wash fluid was purified using Streptactin beads, separated on SDS–PAGE, and gels were stained with Coomassie Brilliant blue. (c) Immunoblot analysis of PS‐SUMO‐AMP1 in apoplast wash fluid. Separated proteins were transferred to a polyvinylidene difluoride membrane (PVDF) and probed with a monoclonal rat anti‐HA antibody (1:1000) and goat anti‐rat IgG (1:4000) to detect PS‐bdSUMO^Eu1^‐AMP1 (∼64.9 kDa). (d) Reversed‐phase liquid chromatography separation of peptides. The lyophilized extract, after SUMO protease cleavage, was separated on a C8 column for a 60‐min run reaction. (e) Tricine–SDS–PAGE analysis of peptide. Peptide separated using RP‐HPLC (dual absorbance at 215/280 nm) was analysed on 12% tricine‐SDS–PAGE and stained with Coomassie Brilliant blue solution containing 40% methanol and 4% formaldehyde. (f) Mass spectrometry sequencing of peptide. The MS/MS spectrum of plant‐purified AMP1 and the amino acid sequence of the peptide is shown with observed *b* ions (represent product when the charge is retained on the N‐terminus) and *y* ions (represent product when the charge is retained on the C‐terminus) mapped.

To facilitate active secretion of AMPs into the apoplast and evaluate the efficiency of the peptide signal in plant cells, we used a codon‐optimized sequence of Δ418 to match the preferred codon usage of *N. benthamiana* (Figure 2a). To enhance the solubility of AMPs and potentially mitigate their toxicity towards the plant host, we fused AMP1 with a mutated SUMO domain as described previously (Chaudhary *et al*., 2023). The SUMO domain was adapted from the grass *B. distachyon* and contains mutations at the SUMO‐protease interacting position, rendering it resistant to degradation from the intracellular plant‐specific SUMO protease and facilitating the release of the AMP without leaving additional residues (Vera Rodriguez *et al*., 2019). Moreover, to retrieve the AMP bearing the apoplast secretory signal peptide sequence and SUMO domain from the apoplast extract, we positioned a Twin‐Strep tag sequence adjacent to the signal sequence in the vector backbone (Figure 2a). Linker sequences were introduced on both sides to enable the independent movement of the tag sequence to bind to its cognate Streptactin beads, facilitating the capture of Strep‐tagged AMP.

To transiently express the AMP, we used a combination of robust synthetic and wild‐type gene expression elements that outperform the current standards as described previously (Peyret *et al*., 2019): the 35S promoter preceded with an enhancer from 35S promoter region (e35S), a synthetic 5′UTR (5S0), and the wild‐type 3′UTR of CPMV. The entire suite of expression modulators, including the Δ418‐SUMO‐AMP1 construct, was introduced into the backbone of the binary vector pMDC43, which has been modified to eliminate native gene regulatory sequences, leaving only the aminoglycoside phosphotransferase gene conferring resistance to kanamycin within the T‐DNA. In addition, we used *p19* (also known as *CDKN2D*) to suppress post‐transcriptional gene silencing, further enhancing gene expression levels.

We expressed Strep‐tagged Δ418‐SUMO‐AMP1 in *N. benthamiana* in a greenhouse using *A. tumefaciens*‐mediated transient protein expression. Leaves were processed 5 days after infiltration to harvest the protein from the apoplast and the resulting apoplastic fluid was processed to purify the AMP.

### Detergent‐free AMP purification

Our previously established protocol for obtaining pure and homogenous recombinant AMPs from the cytosol of plants necessitated a multi‐step process that progressively lowered the peptide concentration at each step (Chaudhary *et al*., 2023). Additionally, we had to supplement the buffer with detergents and various additives; however, it is crucial to eliminate these components from the final peptide product, since they could potentially interfere with the peptide's biological activity. In this study, we performed extractions using detergent‐free and additive‐free buffers, with reduced downstream processing and achieved high peptide yields without compromising purity. To extract the apoplastic fluid containing the soluble AMPs, we performed vacuum‐based infiltration followed by centrifugation.

The resulting apoplastic fluid contained soluble proteins and cellular debris. To purify the AMP, we performed affinity chromatography with Strep beads, which resulted in the recovery of SUMO‐AMP1 protein from the soluble phase. SUMO‐AMP1 was not sequestered into the cellular debris (as observed with hydrophobic peptides), indicating that the fusion to SUMO improves the solubility of the AMPs, thus obviating the need for solubilizing agents and detergents. Furthermore, we noted that expression of SUMO‐AMP1 was readily detectable by Coomassie brilliant blue staining (Figure 2b) and immunoblotting (Figure 2c; Figure S1).

After affinity purification, AMP1 was released from the Strep beads by cleaving the fusion proteins using our previously described, in‐house, recombinantly produced, sentrin‐specific protease (SENP^EuH^), an orthogonal enzyme that hydrolyzes the peptide bond at the C terminus of the modified SUMO^Eu1^ domain. We performed protein cleavage in the absence of detergents and solubilizing agents, and we noted that the exclusion of these additives did not reduce the enzyme's cleavage efficiency. However, peptides with isoelectric points close to the pH of the buffer tend to precipitate, and for such peptides, the inclusion of detergent is crucial to prevent their precipitation.

The released AMP1 peptide was collected in the presence of dithiothreitol (DTT) to prevent aggregation and subsequently subjected to lyophilization. The lyophilized peptide at this stage contains salt and other impurities, which were removed using reversed‐phase high‐performance liquid chromatography. The peptide was eluted between 32 and 35 min (Figure 2d), and analysis of the fraction on 12% tricine–SDS–PAGE gels revealed a single peptide band migrating closely with the lowest protein ladder of 1.7 kDa after staining with Coomassie brilliant blue (Figure 2e). Peptide quantification was performed using the highly sensitive bicinchoninic acid (BCA) assay, as the short length of peptides could lead to variability in absorption by aromatic residues at 280 nm. The concentration of the peptide was 2.9 mg per 20 g of leaf material, and the sequence was confirmed through mass spectrometry sequencing (Figure 2f).

### The plant‐purified peptide retains antibacterial activity

**Figure 3:**
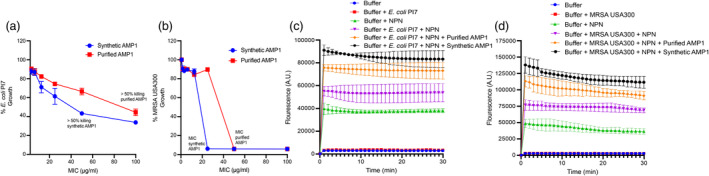
Antibacterial activity of plant‐purified AMP1. (a and b) Bacterial growth inhibition assay in the presence of plant‐purified and synthetic AMP1. Nearly 10^6^ cells colony‐forming units (CFU)/mL of *E. coli* PI7 (grown in LB containing 8 μg mL^−1^ meropenem) and MRSA USA300 (grown in tryptic soy broth containing 10 μg mL^−1^ chloramphenicol) were treated with 100–1.56 μg mL^−1^ of both plant‐purified and synthetic AMP1 in cation‐adjusted Mueller‐Hinton broth for 24 h. Bacterial growth inhibition was measured at OD_600_ using a TECAN Infinite 200 PRO series. Two independent experiments were performed in triplicate, and the data are represented as mean ± SD. (c and d) NPN assay for outer‐membrane permeabilization of *E. coli* PI7 and MRSA USA300 induced by synthetic and plant‐purified AMPs. Profiles show a rapid increase in fluorescence emission intensity, followed by a slow decay. Measurements were conducted on a white, 96‐well plate using a TECAN Infinite 200 PRO series with excitation and emission wavelengths set to 340 and 405 nm, respectively, following the experimental procedure outlined in the Materials and Methods. Two independent experiments were performed in triplicate, and the data are represented as mean ± SD.

After establishing the purification procedure for SUMO‐fused AMPs, we next investigated whether the plant‐purified AMP retained function. To this end, we assessed the antimicrobial activity of the purified peptide in parallel with the synthetic one against clinically relevant pathogens, specifically *E. coli* PI7 (New Delhi metallo‐β‐lactamase‐positive strain isolated from sewage water) and *S. aureus* (MRSA) USA300 (clinically isolated from a patient), which play a significant role in infectious diseases and are listed on the World Health Organization's watchlist. Interestingly, purified AMP1 displayed >50% of *E. coli* PI7 killing at a concentration exceeding 50 μg mL^−1^, while the synthetic peptide achieved similar killing at a concentration = 50 μg mL^−1^ (Figure 3a). Similarly, plant‐purified AMP1 showed a higher MIC (50 μg mL^−1^) compared to its synthetic counterpart (MIC = 25 μg mL^−1^) against MRSA USA300 as well (Figure 3b). This translates to purified AMP1 having a higher MIC compared to synthetic AMP1, which could be attributed to the lack of amidation in the plant‐purified peptide, making it more susceptible to proteolytic degradation.

We further used the fluorescent probe NPN to assess the peptide‐induced outer membrane permeabilization. In an aqueous environment, NPN exhibits weak fluorescence and is only permeable to the outer membrane (Arque *et al*., 2022). Upon interaction with the lipid environment of compromised outer membranes, the probe emits fluorescence at higher intensity, indicating that the peptide has induced membrane permeabilization (Arque *et al*., 2022). The NPN assay demonstrated that the plant‐purified AMP1 permeated the outer membrane of MRSA USA300 and *E. coli* PI7 (Figure 3c,d), similar to the synthetic AMP1, indicating that plant‐purified AMP1 indeed destabilized the membrane of bacterial cells and displayed a killing mechanism similar to that of its synthetic counterpart.

### The plant‐purified peptide exhibits low toxicity in a human primary dermal cell line

**Figure 4:**
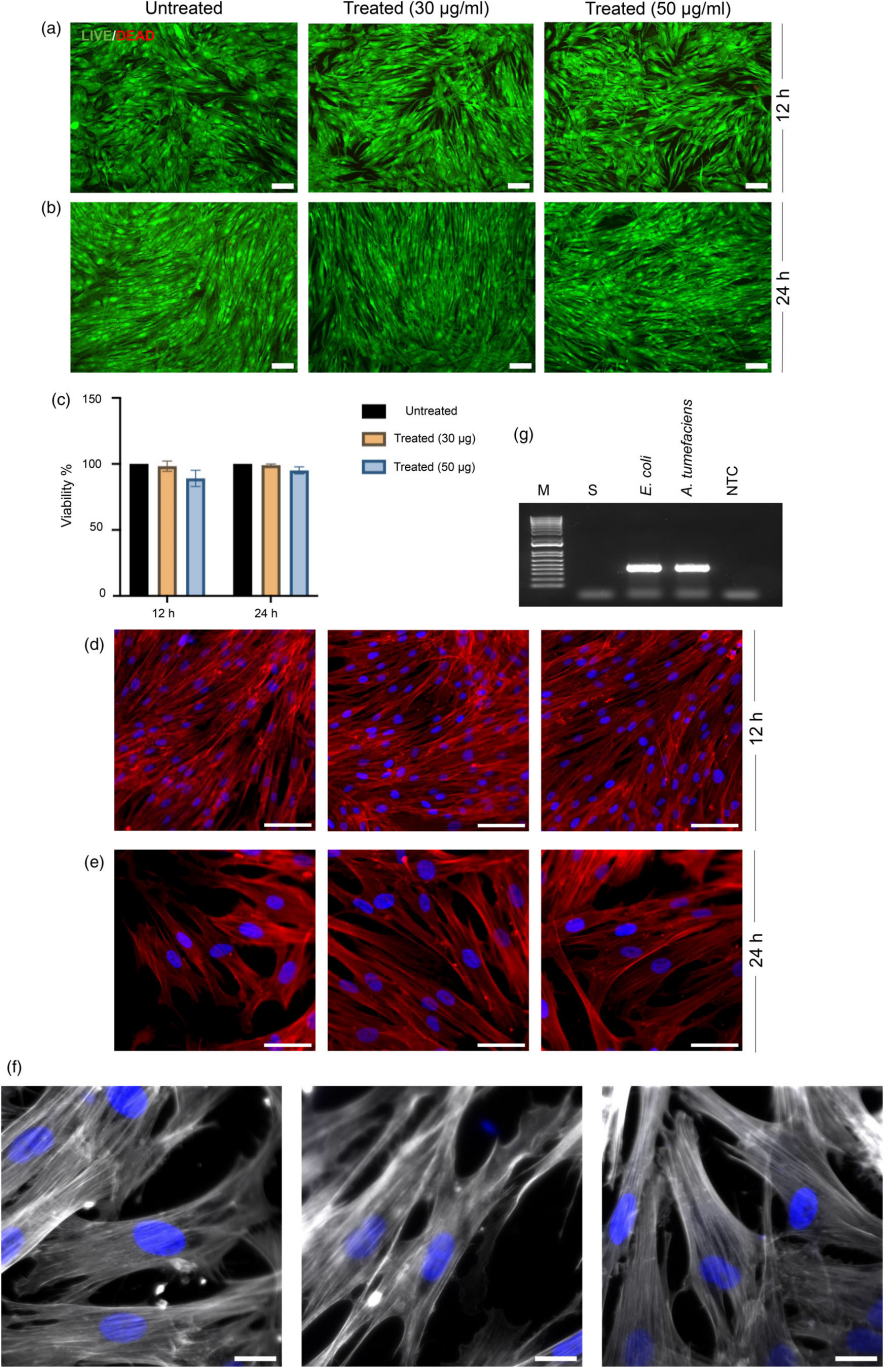
Biocompatibility assessment of purified AMP1 on human dermal fibroblasts in terms of HDF cell viability, proliferation and metabolic activity. (a and b) Live/dead fluorescence staining of human dermal fibroblasts (HDFs) at 12 (a) and 24 h (b) post‐treatment with peptide concentrations of 30 and 50 μg mL^−1^. Untreated HDFs were used as a control. Live cells show green fluorescence, while dead cells have red fluorescence. Scale bars, 100 μm. (c) The metabolic index of treated and untreated HDFs measured by directly estimating the ATP levels using a luciferase‐based CellTiter‐Glo reagent. Statistical significance was calculated using a standard two‐tailed paired t‐test (*n* = 3 biological replicates). (d) Confocal microscopy images of HDFs after cytoskeleton staining of actin fibres (red) and nuclei (blue) at 12 h. Scale bars, 100 μm. (e) The organization of F‐Actin stress fibres within the HDF cells at 24 h. Scale bars, 50 μm. (f) Close‐ups of F‐Actin stress fibres within untreated and treated HDF cells at 24 h of treatment. Scale bars, 20 μm. Three independent microscopy experiments were performed with similar results. (g) Analysis of the purified peptide sample using polymerase chain reaction (PCR) to identify the presence of any bacterial‐specific 16S rRNA sequence. Positive controls consisted of whole genomic DNA from *E. coli* MG1655 and *A. tumefaciens* GV3101. The labelling is as follows: M for 1 kb Plus ladder, S for peptide sample and NTC for negative control.

Next, we tested the purified peptide for cytotoxicity against a human primary dermal cell line. The primary dermal fibroblast was used as a proxy to analyse the toxicity of peptides in contact with the skin since fibroblasts are the most common type of cell found in the dermis, and it is imperative for the drug to demonstrate low toxicity in humans to obtain approval from the U.S. Food and Drug Administration. The peptide concentrations used in the cytotoxicity analysis were 30 and 50 μg mL^−1^ because the minimal inhibitory concentrations of the peptide against a previously tested panel of *Enterococcus faecium*, *S. aureus*, *Klebsiella pneumoniae*, *Acinetobacter baumannii*, *Pseudomonas aeruginosa* and Enterobacter species (ESKAPE) pathogens were below 50 μg mL^−1^ (Chaudhary *et al*., 2023). The LIVE/DEAD viability assay for HDFs revealed that most of the cells remained viable in the untreated and treated groups with 30 and 50 μg mL^−1^ of the peptide. This is evidenced by the predominant presence of green fluorescence‐emitting cells through their interaction with Calcein, signifying the integrity of cell membranes. (Figure 4a,b). This suggests that the peptide concentrations used (30 and 50 μg mL^−1^) were not cytotoxic to HDFs within the tested time frames of 12 and 24 h. Similarly, we assessed the cellular metabolic activity of the HDFs with the CellTiter‐Glo 3D Cell Viability assay and found no significant difference in ATP release between peptide‐treated and control groups (Figure 4c), indicating that the peptide's presence does not adversely affect the metabolic function of HDFs. Moreover, the structural analysis of the actin cytoskeleton, facilitated by rhodamine‐phalloidin staining, demonstrated no noticeable alterations in the organization of actin filaments between treated and untreated cells. The actin stress fibres appeared consistent across all samples, suggesting that the peptide treatment did not disrupt the cytoskeletal architecture (Figure 4d–f). However, mild cytotoxicity is observed at a concentration of 100 μg mL^−1^ (2‐ and 4‐fold higher than its MIC), with 65% of cells retaining their viability, thus proving a pronounced therapeutic window (Figure S2). It is to be expected that there will be a weaker interaction between highly cationic and amphipathic peptides, such as AMP1, with eukaryotic membranes compared to bacterial membranes, due to the higher hydrophobicity and slightly negative charge of eukaryotic membranes (Casares *et al*., 2019; Glukhov *et al*., 2005).

Since we used *A. tumefaciens* to induce transient expression for peptide production in *N. benthamiana*, we analysed the endotoxin concentration in peptide preparation, which was found to be at 0.0896 EU mL^−1^, significantly below the threshold tolerated by the U.S. Food and Drug Administration for an injectable drug. This suggests the exceptionally low presence (or absence) of bacterial toxin in the purified peptide sample. We further estimated the bacterial burden by amplifying the conserved domain of the 16S rRNA sequence of bacterial species, revealing no trace of bacterial DNA in the peptide sample (Figure 4g). Taken together, our results indicate that the purified peptide was not only biocompatible with HDFs, as they do not compromise cell viability, metabolic activity or cytoskeletal organization but also contained a low level of endotoxin. These findings are promising for further applications of these plant‐produced peptides in biomedical and therapeutic contexts.

### *In vivo* antimicrobial activity of purified peptide in a mouse model

**Figure 5:**
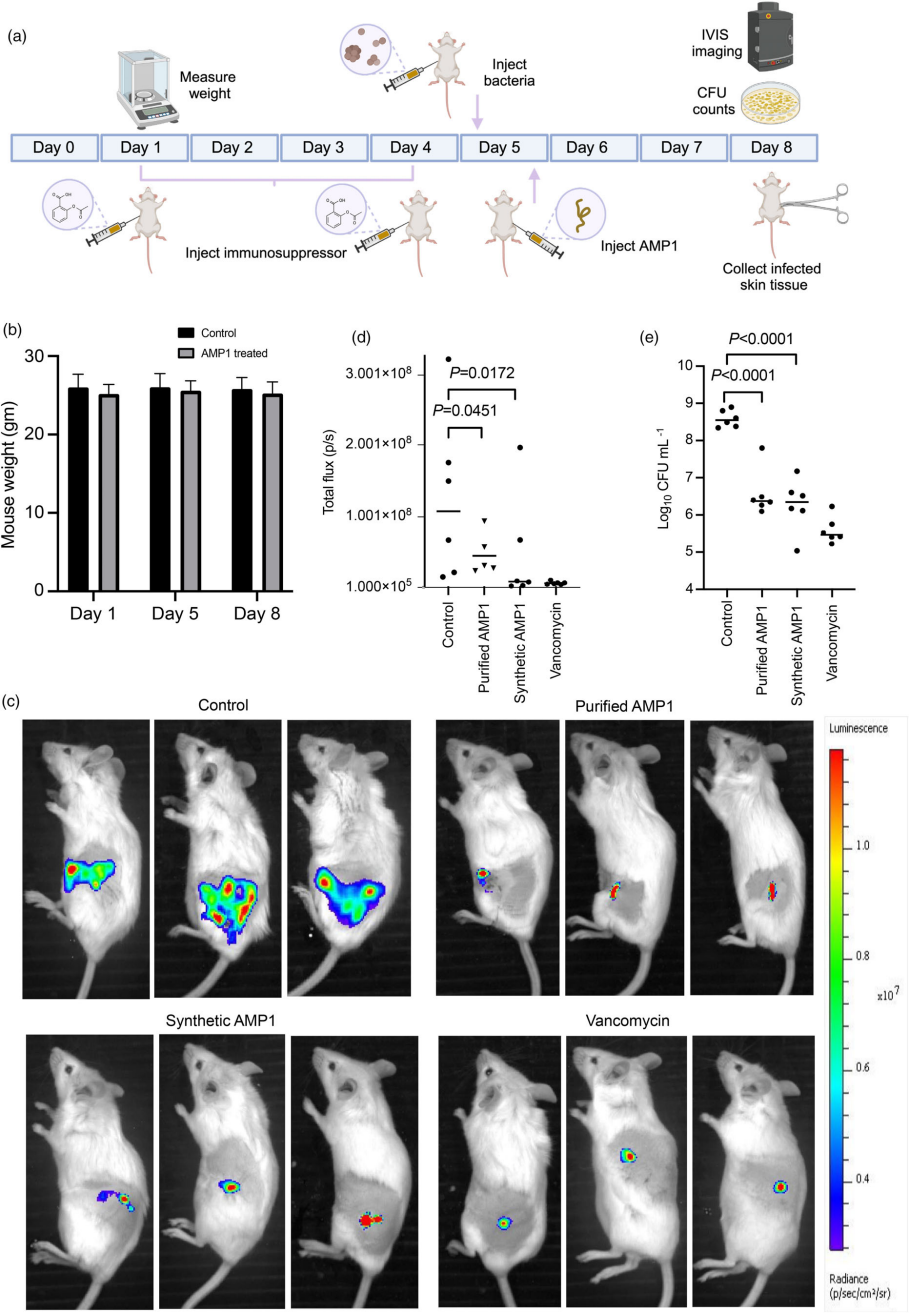
Anti‐bacterial activity of purified AMP1 against a bacterial pathogen in a mouse infection model. (a) Schematic representation of the skin abscess model used to assess the anti‐bacterial activity of purified AMP1 (*n* = 6 vehicle‐treated group; *n* = 6 purified AMP1‐treated group; *n* = 6 synthetic AMP1‐treated group and *n* = 6 vancomycin hydrochloride‐treated group). (b) Weight of the mice was consistently monitored over the course of the 1‐, 5‐ and 8‐day experiments to eliminate the possibility of any potential toxic effects from the purified peptide. Statistical analysis was performed using a standard two‐tailed paired *t*‐test. (c) Representative IVIS images of the whole body of mice acquired 72 h after infection with luminescent MRSA tagged with the lux operon, using an IVIS Spectrum imaging system. For the treated group, the purified peptide was injected 60 min after bacterial inoculation. Synthetic AMP1 at a dose of 7 mg Kg^−1^ and vancomycin hydrochloride at a dose of 150 mg Kg^−1^ were used as controls. (d) Quantification of luminescence signal expressed as total flux (photons/s) in the region of interest (ROI) of bacterial load of the mice. The final ROI was calculated by subtracting the average background ROI from the measurement ROI. (e) Treatment with the peptide leads to decreased MRSA USA300 bacterial counts compared with the control group (not treated with the peptide). Statistical significance in d and e was calculated using a one‐way ANOVA (Tukey's multiple comparison tests), *P < 0.05, **P < 0.01, ***P < 0.001, ****P < 0.0001.

The pathogenic bacterium MRSA USA300 is responsible for skin and soft tissue infections, prosthetic joint infections, and necrotizing pneumonia (Chanin *et al*., 2011). This bacterium has acquired resistance to existing antibiotics (McDougal *et al*., 2010; Tenover and Goering, 2009), and AMPs are promising candidates for treating these infections. To test whether the plant‐purified peptides retain their antimicrobial activity *in vivo*, we tested them against MRSA in a mouse model (Figure 5a). To evaluate the *in vivo* toxicity, mice were weighed pre‐ and post‐treatment. The monitoring of weight fluctuations served as a crucial measure, with variations of up to 20% considered as a widely deter proxy for distress, morbidity and overall toxicity (Arque *et al*., 2022; Kim *et al*., 2001; Talbot *et al*., 2020). Additionally, we monitored mice for toxicity markers, including itchiness (LaMotte *et al*., 2011; Shimada and LaMotte, 2008), redness (Jamshaid *et al*., 2022) and swelling (Mekonnen *et al*., 2019; Siddique *et al*., 2015). Notably, the tested peptide exhibited no side effects or toxicity *in vivo*, as evidenced by the absence of significant changes in the body weight of treated mice compared to untreated counterparts (Figure 5b). We monitored skin infection by administering a 1 × 10^7^ CFU mL^−1^ solution of MRSA USA300 on the flank of mice and treated the abscess with a single dose of purified AMP1 (7 mg kg^−1^). At 3 days after the treatment, the purified peptide showed potent bactericidal activity *in vivo* (*P =* 0.0451) similar to that of its synthetic counterpart (*P* = 0.0172) (Figure 5c,d), demonstrating the effectiveness of plant‐purified AMP1. While the treatment did not completely sterilize the infection, it reduced bacterial loads by three orders of magnitude (*P* = 0.0001) (Figure 5e). Interestingly, we observed that mice treated with the peptide exhibited lower disease severity induced by bacterial infection compared to the control group, and this can be attributed to the dual properties of AMP1, which possesses both anti‐inflammatory and antibacterial functions, thereby suppressing inflammation and hindering bacterial growth (Wu *et al*., 2017). Overall, our *in vivo* results highlight the microbicidal efficacy of the plant‐purified peptide under physiological conditions and suggest that plants can serve as a promising platform for antibacterial agent production.

### Techno‐economic analysis of AMP1 production in plants

We conducted a simulation‐based techno‐economic analysis to predict the final cost of AMP1 produced in *N. benthamiana* and secreted to the apoplast, and further investigated whether this system reduces the downstream processing costs. For the analysis, we used the previously developed base‐case scenario assuming 10 000 kg *N. benthamiana* plant fresh weight (FW) containing 2 kg AMP1 harvested from the apoplast with an expression level of 200 mg per kg plant FW. The assumptions and results developed in SuperPro Designer® 13.0 software were used to compute the economic viability of the described process. Table 1 provides an overview of the total operating expenses, classified separately for both upstream and downstream components. In upstream operations, the largest components are facility‐dependent costs ($3 368 000) followed by utilities ($1 901 000), whereas in the downstream operations, the largest components are facility‐dependent costs ($5 234 000) followed by materials ($2 850 271). Overall, the upstream components represent nearly 46.08% of the total production cost of AMP1, while the downstream components represent 53.9%. This still signifies a significant reduction in downstream processing from our previous base case scenario, where downstream processing accounted for 68.99% (Chaudhary *et al*., 2023).

**Table 1:** Summary of upstream and downstream production costs

Process component	Upstream	Downstream	Total
Materials	970 328	2 850 271	3 820 599
Facility‐dependent costs	3 368 000	5 234 000	8 602 000
Labor‐dependent costs (annual)	1 484 421	1 603 000	3 087 421
Lab QA/QC (annual)	120 000	801 000	921 000
Consumables (annual)	1 243 000	445 357	1 688 357
Utilities (annual)	1 901 000	10 943	1 911 943
Waste treatment (annual)	289 000	26 260	315 260
Total operating expenses excluding depreciation ($/year)	9 431 964	11 423 292	20 855 256
Total operating expenses with depreciation ($/year)	13 204 750	16 707 831	29 912 581
Unit production cost or COGS ($/gram of AMP1)	$31.43	$38.07	$69.5
COGS of a 3‐mg dose of AMP1	$0.09	$0.11	$0.2

Abbreviations: COGS, cost of goods sold; QA, quality assurance; QC, quality control.

The techno‐economic analysis emphasized the use of plant‐produced AMPs as cost‐effective microbicides, given that the traditional production methods of AMPs limit their application due to high costs. Previous evaluation of the techno‐economic analysis of AMPs produced by traditional platforms, such as microbial culture, demonstrated that the total operating cost of bacterially produced AMPs was 42 €/mg (Gaglione *et al*., 2019). Conversely, in comparison, antimicrobials produced from plants by Nomad Biosciences (Halle, Germany) cost between $3.00 and $6.88 per gram (McNulty *et al*., 2020). This suggests that plant‐based protein production remains significantly more cost‐effective, estimated 10–50 times less expensive than *E. coli* fermentation. The overall COGS of our apoplast‐based AMP purification protocol is in alignment with the previous study, and for an AMP1 dose of 3 mg, the per‐dose manufacturing cost is $0.20. However, the protocol necessitates the use of commercial Strep‐Tactin resins, purified protease enzyme and an established RP‐HPLC chromatography infrastructure. This could raise production costs in both developed countries and the Global South, despite the inexpensive labor costs in developing countries.

Most affinity tags are expensive, often necessitating the use of orthogonal protease enzymes to release peptides in their native biological state, thereby increasing the overall production cost (Fong *et al*., 2010). However, there are reports suggesting that the bactericidal activity of AMPs can remain unaffected even without tag removal (Ghidey *et al*., 2020; Hoelscher *et al*., 2022). For instance, one of the studies proposed the use of cost‐effective ELP‐tagged AMPs purification and achieved unprecedented potency in killing bacteria (MIC of 0.3 μM) without removing the affinity tag (Ghidey *et al*., 2020). Although their protocol is simpler and more cost‐effective compared to our apoplast‐based platform, the inclusion of additional purification steps mandated in our platform is to refine the peptide by eliminating any residual amino acids and other potential host‐related impurities that can trigger an immune response upon systemic injection. Moreover, our plant‐produced AMP demonstrated strong antibacterial activity against highly drug‐resistant BSL2 pathogens within the therapeutic window (<50 μM), lacks residual immunogenic amino acids, is highly pure, and holds promise for clinical translation.

In conclusion, SPPS and bacterial systems have already established a niche in the market for producing AMPs. However, plants are considered an ‘emerging’ platform, and with advancements in molecular farming, plants can be genetically engineered for large‐scale and cost‐effective production of AMPs, as demonstrated in this study. Furthermore, plant‐produced AMPs can be utilized as microbicides, as we demonstrated the safety of plant‐produced AMP1 in HDFs and its efficacy in a mouse model, as well as the cost‐effectiveness of our system through techno‐economic analysis. Therefore, our plant‐based AMP production system, involving apoplast secretion of the accumulated peptide, holds potential for clinical testing in humans.

## Competing interests

The authors declare no competing interests.

## Supporting information


**Figure S1.** Apoplast secretion of SUMO‐AMP1.
**Figure S2.** Biocompatibility of plant‐purified AMP1 on human dermal fibroblast cells (HDFs).

## Data Availability

Data sharing is not applicable to this article as no new data were created or analyzed in this study.
