# Supplementary material for: High‐yield, plant‐based production of an antimicrobial peptide with potent activity in a mouse model
Source: Plant Biotechnol J. 2024 Sep 12;22(12):3392–405. doi: 10.1111/pbi.14460 (PMC11606426; doi:10.1111/pbi.14460)
Supplement: Supplementary file 1 — Figure S1. Apoplast secretion of SUMO‐AMP1. Figure S2. Biocompatibility of plant‐purified AMP1 on human dermal fibroblast cells (HDFs). [file PBI-22-3392-s001.docx]

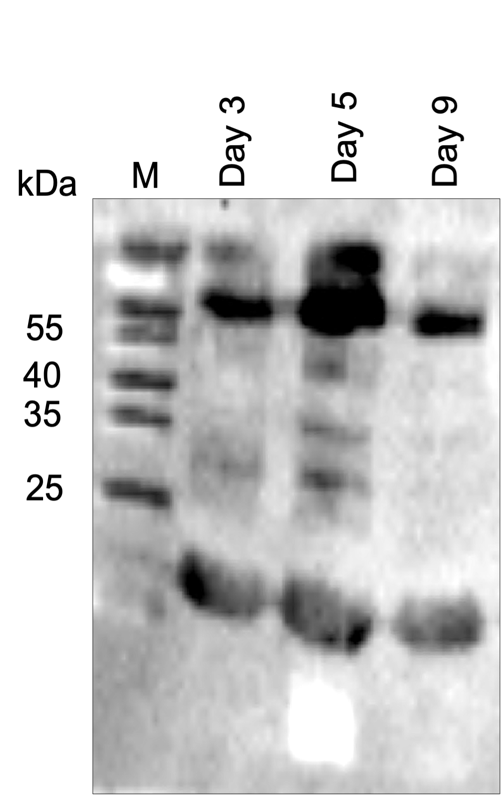


**Supplementary Figure 1:** **Apoplast secretion of SUMO-AMP1.** The *Agrobacterium* GV3101 bacterial strain carrying binary plasmid encoding for PS-SUMO-AMP1 was infiltrated into *N. benthamiana*. Apoplast fluid was collected on days 3, 5, and 9 post-infiltration and analyzed by immunoblotting.

**Supplementary Figure 2:** **Biocompatibility of plant-purified AMP1 on human dermal fibroblast cells (HDFs)**. Primary HDFs were cultured in presence of varying concentrations of purified AMP1 (200, 100, 50, 25, 12.5 μg mL^-1^), control (DMEM treated) and positive control (0.1% Triton X-100) and cell viability was assayed using the CellTiter-Glo 3D reagent based on ATPs quantification after 24 h. Two independent experiments were performed in triplicate, and the data are represented as mean ± SD.

| **Name** | **Sequence (5’-3’)** | **Experimental description** |
| --- | --- | --- |
| FP gBlock root | TCTCTGGTCTCACTCTATGATGAGAGC | PCR amplification of gBlock (contain BsaI site) |
| RP gBlock root | GAGCGGGTCTCAAGCGTTAACCC | PCR amplification of gBlock (contain BsaI site) |
| Oligo root top | TTTAAGAGACGCAACCACAACGCTCTAACGCAATCAATCTACATTATATTAAACGT | Overlapping oligos (containing 5’ BsmBI and 3’ BsaI overhangs |
| Oligo root bot | AGAGACGTTTAATATAATGTAGATTGATTGCGTTAGAGCGTTGTGGTTGCGTCTCT | Overlapping oligos (containing 5’ BsmBI and 3’ BsaI overhangs |
| Phaseolin F | ATAGGCGCGCCATTGTTAGAGGGAGCTCTGAT | Amplification of pREAQ promoter and gBlock (contain AscI site; for cloning into Δ35pMDC43 vector) |
| Phaseolin R | TTTGGAGTAGGGGGGTACCTGT | Amplification of pREAQ promoter and gBlock |
| Seq PR1 | CCAGCAACAGGAGTGGAACCCTTGC | Sequencing of construct |
| Seq PR2 | CTGTTGCTGGGAATTCTTTTCCTGGCA | Sequencing of construct |
| Seq PR3 | GCTGTCCCTCCTCTTCAAACAGAA | Sequencing of construct |
| Seq PR4 | GGACAGCAAGAGGGAGTGATTGTGAAC | Sequencing of construct |
| Seq PR5 | GCTGTTCCTGTTGGTGATGGTGTGCA | Sequencing of construct |
| Seq PR6 | CAGCAAAAGGGAAGAAAGGGTGGTGGC | Sequencing of construct |
| Seq PR7 | GCACAGAAAACCGCTCACCAAAC | Sequencing of construct |
| Seq PR8 | CTGTGCTCAGAGTGTGTTTATTTTATGTA | Sequencing of construct |
| 16s rRNA F | TCCTACGGGAGGCAGCAGT | PCR amplification of 16s rRNA |
| 16s rRNA R | GGACTACCAGGGTATCTAATCCTGTT | PCR amplification of 16s rRNA |

gBlock

Signal sequence linker sequences Twin Strep tag sequences HA sequences linker sequences Mutated SUMO domain AMP1 peptide sequence

TCTCTGGTCTCACTCTATGATGAGAGCAAGGGTTCCACTCCTGTTGCTGGGAATTCTTTTCCTGGCATCACTTTCTGCCTCATTTGCCACTTCACTCCGGGAGGAGGAAGAGAGCCAAGATAACCCCTTCTACTTCAACTCTGACAACTCCTGGAACACTCTATTCAAAAACCAATATGGTCACATTCGTGTCCTCCAGAGGTTCGACCAACAATCCAAACGACTTCAGAATCTTGAAGACTACCGTCTTGTGGAGTTCAGGTCCAAACCCGAAACCCTCCTTCTTCCTCAGCAGGCTGATGCTGAGTTACTCCTAGTTGTCCGTAGTGGGAGCGCCATACTCGTCTTGGTGAAACCTGATGATCGCAGAGAGTACTTCTTCCTTACGAGCGATAACCCGATATTCTCTGATCACCAGAAAATCCCTGCAGGAACCATTTTCTATTTGGTTAACCCTGACCCCAAAGAGGATCTCAGAATAATCCAACTCGCCATGCCCGTTAACAACCCTCAGATTCATGAATTTTTCCTATCTAGCACAGAAGCCCAACAATCCTACTTGCAAGAGTTCAGCAAGCATATTCTAGAGGCCTCCTTCAATAGCAAATTCGAGGAGATCAACAGGGTTCTGTTTGAAGAGGAGGGACAGCAAGAGGGAGTGATTGTGAACATTGATTCTGAACAAATTAAGGAACTGAGCAAACATGCAAAATCTAGTTCAAGGAAATCCCTTTCCAAACAAGATAACACAATTGGAAACGAATTTGGAAACCTGACTGAGAGGACCGATAACAGTTTGAATGTGTTAATCAGTTCTATAGAGATGGAAGAGGGAGCTCTTTTTGTGCCACACTACTATTCTAAGGCCATTGTTATACTAGTGGTTAATGAAGGAGAAGCACATGTTGAACTTGTTGGCCCAAAAGGAAATAAGGAAACCTTGGAAATTGAGAGCTACAGAGCTGAGCTTTCTAAAGACGATGTATTTGTAATCCCAGCAGCATATCCAGTTGCCATCAAGGCTACCTCCAACGTGAATTTCTTTGGTTTCGGTATCAATGCTAATAACAACAATAGGAACCTCCTTGCAGGTAAGACGGACAATGTCATAAGCAGCATCGGTAGAGCTCTGGACGGTAAAGACGTGTTGGGGCTTACGTTCTCTGGGTCTGGTGACGAAGTTATGAAGCTGATCAACAAACAGAGTGGATCGTACTTTGTGGATGCACACCATCACCAACAGGAACAGCAAAAGGGAAGAAAGGGTGGTGGCGGGGGATCTGGAGGTGGTGGAAGCTGGTCACATCCACAATTTGAGAAAGGAGGCGGATCCGGGGGGGGAAGCGGCGGTTCTGCTTGGTCACATCCTCAATTCGAAAAGTACCCCTACGACGTTCCTGATTACGCTTACCCTTACGATGTACCGGACTACGCATATCCGTACGATGTCCCAGATTACGCCGGTGGAGGTGGGAGCGGGGGCGGAGGGAGCCATATTAATTTGAAGGTTAAGGGACAAGATGGGAATGAGGTATTTTTCCGTATTAAACGGTCTACACAGTTGAAGAAGCTTATGAACGCCTACTGTGATAGACAGTCTGTTGACATGAAGGCAATAGCATTTCTCTTTAAGGGACGTAGATTGAGAGCTGAGAGAACGCCAGATGAACTTGAAATGGAGGACGGGGATGAGATTGACGCTATGTTGCATCAGACTGGTGGTGTACAGAGATGGTTGATAGTATGGAGAATAAGAAAGGGATAACGCTTGAGACCCGCTC

**AMP1 construct in pMDC43 backbone**

ATCCTCGAGCTGATCACTGAGCATCGTTGAAGATGCCTCTGCCGACAGTGGTCCCAAAGATGGACCCCCACCCACGAGGAGCATCGTGGAAAAAGAAGACGTTCCAACCACGTCTTCAAAGCAAGTGGATTGATGTGATATCTCCACTGACGTAAGGGATGACGCACAATCCCACTATCCTTCTTAATTAAGGAAACCTCCTCGGATTCCATTGCCCAGCTATCTGTCACTTTATTGAGAAGATAGTGGAAAAGGAAGGTGGCTCCTACAAATGCCATCATTGCGATAAAGGAAAGGCCATCGTTGAAGATGCCTCTGCCGACAGTGGTCCCAAAGATGGACCCCCACCCACGAGGAGCATCGTGGAAAAAGAAGACGTTCCAACCACGTCTTCAAAGCAAGTGGATTGATGTGATATCTCCACTGACGTAAGGGATGACGCACAATCCCACTATCCTTCGCAAGACCCTTCCTCTATATAAGGAAGTTCATTTCATTTGGAGAGGTTTAAGAGACGCAACCACAACGCTCTAACGCAATCAATCTACATTATATTAAACGTCTCTATGATGAGAGCAAGGGTTCCACTCCTGTTGCTGGGAATTCTTTTCCTGGCATCACTTTCTGCCTCATTTGCCACTTCACTCCGGGAGGAGGAAGAGAGCCAAGATAACCCCTTCTACTTCAACTCTGACAACTCCTGGAACACTCTATTCAAAAACCAATATGGTCACATTCGTGTCCTCCAGAGGTTCGACCAACAATCCAAACGACTTCAGAATCTTGAAGACTACCGTCTTGTGGAGTTCAGGTCCAAACCCGAAACCCTCCTTCTTCCTCAGCAGGCTGATGCTGAGTTACTCCTAGTTGTCCGTAGTGGGAGCGCCATACTCGTCTTGGTGAAACCTGATGATCGCAGAGAGTACTTCTTCCTTACGAGCGATAACCCGATATTCTCTGATCACCAGAAAATCCCTGCAGGAACCATTTTCTATTTGGTTAACCCTGACCCCAAAGAGGATCTCAGAATAATCCAACTCGCCATGCCCGTTAACAACCCTCAGATTCATGAATTTTTCCTATCTAGCACAGAAGCCCAACAATCCTACTTGCAAGAGTTCAGCAAGCATATTCTAGAGGCCTCCTTCAATAGCAAATTCGAGGAGATCAACAGGGTTCTGTTTGAAGAGGAGGGACAGCAAGAGGGAGTGATTGTGAACATTGATTCTGAACAAATTAAGGAACTGAGCAAACATGCAAAATCTAGTTCAAGGAAATCCCTTTCCAAACAAGATAACACAATTGGAAACGAATTTGGAAACCTGACTGAGAGGACCGATAACAGTTTGAATGTGTTAATCAGTTCTATAGAGATGGAAGAGGGAGCTCTTTTTGTGCCACACTACTATTCTAAGGCCATTGTTATACTAGTGGTTAATGAAGGAGAAGCACATGTTGAACTTGTTGGCCCAAAAGGAAATAAGGAAACCTTGGAAATTGAGAGCTACAGAGCTGAGCTTTCTAAAGACGATGTATTTGTAATCCCAGCAGCATATCCAGTTGCCATCAAGGCTACCTCCAACGTGAATTTCTTTGGTTTCGGTATCAATGCTAATAACAACAATAGGAACCTCCTTGCAGGTAAGACGGACAATGTCATAAGCAGCATCGGTAGAGCTCTGGACGGTAAAGACGTGTTGGGGCTTACGTTCTCTGGGTCTGGTGACGAAGTTATGAAGCTGATCAACAAACAGAGTGGATCGTACTTTGTGGATGCACACCATCACCAACAGGAACAGCAAAAGGGAAGAAAGGGTGGTGGCGGGGGATCTGGAGGTGGTGGAAGCTGGTCACATCCACAATTTGAGAAAGGAGGCGGATCCGGGGGGGGAAGCGGCGGTTCTGCTTGGTCACATCCTCAATTCGAAAAGTACCCCTACGACGTTCCTGATTACGCTTACCCTTACGATGTACCGGACTACGCATATCCGTACGATGTCCCAGATTACGCCGGTGGAGGTGGGAGCGGGGGCGGAGGGAGCCATATTAATTTGAAGGTTAAGGGACAAGATGGGAATGAGGTATTTTTCCGTATTAAACGGTCTACACAGTTGAAGAAGCTTATGAACGCCTACTGTGATAGACAGTCTGTTGACATGAAGGCAATAGCATTTCTCTTTAAGGGACGTAGATTGAGAGCTGAGAGAACGCCAGATGAACTTGAAATGGAGGACGGGGATGAGATTGACGCTATGTTGCATCAGACTGGTGGTGTTAGGCTTATTGTTGCCGTTAGGATTTGGAGGAGGGGTTAACGCTCTGGTTTCATTAAATTTTCTTTAGTTTGAATTTACTGTTATTCGGTGTGCATTTCTATGTTTGGTGAGCGGTTTTCTGTGCTCAGAGTGTGTTTATTTTATGTAATTTAATTTCTTTGTGAGCTCCTGTTTAGCAGGTCGTCCCTTCAGCAAGGACACAAAAAGATTTTAATTTTATTGATCGTTCAAACATTTGGCAATAAAGTTTCTTAAGATTGAATCCTGTTGCCGGTCTTGCGATGATTATCATATAATTTCTGTTGAATTACGTTAAGCATGTAATAATTAACATGTAATGCATGACGTTATTTATGAGATGGGTTTTTATGATTAGAGTCCCGCAATTATACATTTAATACGCGATAGAAAACAAAATATAGCGCGCAAACTAGGATAAATTATCGCGCGCGGTGTCATCTATGTTACTAGATC

**bdSENP^H^ enzyme sequence for expression in *E. coli***

ATCCTCGAGCTGATCACTGAGCATCGTTGAAGATGCCTCTGCCGACAGTGGTCCCAAAGATGGACCCCCACCCACGAGGAGCATCGTGGAAAAAGAAGACGTTCCAACCACGTCTTCAAAGCAAGTGGATTGATGTGATATCTCCACTGACGTAAGGGATGACGCACAATCCCACTATCCTTCTTAATTAAGGAAACCTCCTCGGATTCCATTGCCCAGCTATCTGTCACTTTATTGAGAAGATAGTGGAAAAGGAAGGTGGCTCCTACAAATGCCATCATTGCGATAAAGGAAAGGCCATCGTTGAAGATGCCTCTGCCGACAGTGGTCCCAAAGATGGACCCCCACCCACGAGGAGCATCGTGGAAAAAGAAGACGTTCCAACCACGTCTTCAAAGCAAGTGGATTGATGTGATATCTCCACTGACGTAAGGGATGACGCACAATCCCACTATCCTTCGCAAGACCCTTCCTCTATATAAGGAAGTTCATTTCATTTGGAGAGGTTTAAGAGACGCAACCACAACGCTCTAACGCAATCAATCTACATTATATTAAACGTCTCTATGATGAGAGCAAGGGTTCCACTCCTGTTGCTGGGAATTCTTTTCCTGGCATCACTTTCTGCCTCATTTGCCACTTCACTCCGGGAGGAGGAAGAGAGCCAAGATAACCCCTTCTACTTCAACTCTGACAACTCCTGGAACACTCTATTCAAAAACCAATATGGTCACATTCGTGTCCTCCAGAGGTTCGACCAACAATCCAAACGACTTCAGAATCTTGAAGACTACCGTCTTGTGGAGTTCAGGTCCAAACCCGAAACCCTCCTTCTTCCTCAGCAGGCTGATGCTGAGTTACTCCTAGTTGTCCGTAGTGGGAGCGCCATACTCGTCTTGGTGAAACCTGATGATCGCAGAGAGTACTTCTTCCTTACGAGCGATAACCCGATATTCTCTGATCACCAGAAAATCCCTGCAGGAACCATTTTCTATTTGGTTAACCCTGACCCCAAAGAGGATCTCAGAATAATCCAACTCGCCATGCCCGTTAACAACCCTCAGATTCATGAATTTTTCCTATCTAGCACAGAAGCCCAACAATCCTACTTGCAAGAGTTCAGCAAGCATATTCTAGAGGCCTCCTTCAATAGCAAATTCGAGGAGATCAACAGGGTTCTGTTTGAAGAGGAGGGACAGCAAGAGGGAGTGATTGTGAACATTGATTCTGAACAAATTAAGGAACTGAGCAAACATGCAAAATCTAGTTCAAGGAAATCCCTTTCCAAACAAGATAACACAATTGGAAACGAATTTGGAAACCTGACTGAGAGGACCGATAACAGTTTGAATGTGTTAATCAGTTCTATAGAGATGGAAGAGGGAGCTCTTTTTGTGCCACACTACTATTCTAAGGCCATTGTTATACTAGTGGTTAATGAAGGAGAAGCACATGTTGAACTTGTTGGCCCAAAAGGAAATAAGGAAACCTTGGAAATTGAGAGCTACAGAGCTGAGCTTTCTAAAGACGATGTATTTGTAATCCCAGCAGCATATCCAGTTGCCATCAAGGCTACCTCCAACGTGAATTTCTTTGGTTTCGGTATCAATGCTAATAACAACAATAGGAACCTCCTTGCAGGTAAGACGGACAATGTCATAAGCAGCATCGGTAGAGCTCTGGACGGTAAAGACGTGTTGGGGCTTACGTTCTCTGGGTCTGGTGACGAAGTTATGAAGCTGATCAACAAACAGAGTGGATCGTACTTTGTGGATGCACACCATCACCAACAGGAACAGCAAAAGGGAAGAAAGGGTGGTGGCGGGGGATCTGGAGGTGGTGGAAGCTGGTCACATCCACAATTTGAGAAAGGAGGCGGATCCGGGGGGGGAAGCGGCGGTTCTGCTTGGTCACATCCTCAATTCGAAAAGTACCCCTACGACGTTCCTGATTACGCTTACCCTTACGATGTACCGGACTACGCATATCCGTACGATGTCCCAGATTACGCCGGTGGAGGTGGGAGCGGGGGCGGAGGGAGCCATATTAATTTGAAGGTTAAGGGACAAGATGGGAATGAGGTATTTTTCCGTATTAAACGGTCTACACAGTTGAAGAAGCTTATGAACGCCTACTGTGATAGACAGTCTGTTGACATGAAGGCAATAGCATTTCTCTTTAAGGGACGTAGATTGAGAGCTGAGAGAACGCCAGATGAACTTGAAATGGAGGACGGGGATGAGATTGACGCTATGTTGCATCAGACTGGTGGTGTTAGGCTTATTGTTGCCGTTAGGATTTGGAGGAGGGGTTAACGCTCTGGTTTCATTAAATTTTCTTTAGTTTGAATTTACTGTTATTCGGTGTGCATTTCTATGTTTGGTGAGCGGTTTTCTGTGCTCAGAGTGTGTTTATTTTATGTAATTTAATTTCTTTGTGAGCTCCTGTTTAGCAGGTCGTCCCTTCAGCAAGGACACAAAAAGATTTTAATTTTATTGATCGTTCAAACATTTGGCAATAAAGTTTCTTAAGATTGAATCCTGTTGCCGGTCTTGCGATGATTATCATATAATTTCTGTTGAATTACGTTAAGCATGTAATAATTAACATGTAATGCATGACGTTATTTATGAGATGGGTTTTTATGATTAGAGTCCCGCAATTATACATTTAATACGCGATAGAAAACAAAATATAGCGCGCAAACTAGGATAAATTATCGCGCGCGGTGTCATCTATGTTACTAGATC
